# Functional Verification of the Citrate Transporter Gene in a Wine Lactic Acid Bacterium, *Lactiplantibacillus plantarum*

**DOI:** 10.3389/fbioe.2022.894870

**Published:** 2022-05-09

**Authors:** Xiangke Yang, Lili Zhao, Qiling Chen, Nan Wang, Kan Shi, Shuwen Liu

**Affiliations:** ^1^ College of Enology, Northwest A&F University, Yangling, China; ^2^ College of Food and Bioengineering, Henan University of Animal Husbandry and Economy, Zhengzhou, China; ^3^ Shaanxi Engineering Research Center for Viti-Viniculture, Yangling, China; ^4^ Engineering Research Center for Viti-Viniculture, National Forestry and Grassland Administration, Yangling, China; ^5^ Heyang Experimental and Demonstrational Stations for Grape, Northwest A&F University, Weinan, China; ^6^ Ningxia Helan Mountain’s East Foothill Wine Experiment and Demonstration Station, Northwest A&F University, Yongning, China

**Keywords:** citrate transporter gene, functional verification, lactiplantibacillus plantarum, organic acids, lactic acid bacteria, gene editing

## Abstract

Organic acid metabolism by lactic acid bacteria plays a significant role in improving wine quality. During this process, the uptake of extracellular organic acids by the transporters is the first rate-limiting step. However, up to now, there is very little published research on the functional verification of organic acid transporter genes in wine lactic acid bacteria. In this study, a predicted citrate transporter gene JKL54_04345 (*citP*) by protein homology analysis was knocked out using a CRISPR/Cas9-based gene-editing system, and then complemented using the modified pMG36e vectors in a major wine lactic acid bacterium*, Lactiplantibacillus plantarum* XJ25, to verify its function in citrate metabolism for the first time. The results showed that the gene knockout mutant XJ25-Δ*citP* lost the ability to utilize citric acid, while the gene complement mutant XJ25-Δ*citP*-pMG36ek11-*citP* fully recovered the ability of citric acid utilization. Meanwhile, *citP* knockout and complement barely affected the utilization of l-malic acid. These indicated that *citP* in *L. plantarum* functioned as a citrate transporter and was the only gene responsible for citrate transporter. In addition, two modified plasmid vectors used for gene supplement in *L. plantarum* showed distinct transcription efficiency. The transcription efficiency of *citP* in XJ25-Δ*citP*-pMG36ek11-*citP* mutant was 4.01 times higher than that in XJ25-Δ*citP*-pMG36ek-*citP* mutant, and the utilization rate of citric acid in the former was 3.95 times higher than that in the latter, indicating that pMG36ek11 can be used as a high-level expression vector in lactic acid bacteria.

## Introduction

Many lactic acid bacteria (LAB) species, known for their positive effects on human health, are of great importance in the food industry (Judkins et al., 2020). The excess retained acid after wine fermentation could lead to the excessively sour taste of the wine products, which greatly limits wine quality. Malolactic fermentation (MLF) induced by wine LAB is generally considered an indispensable process for decreasing the total acidity of wine and brewing excellent wine (Meng et al., 2021). *Lactiplantibacillus plantarum,* which often has a broad range of natural habitats, is one of the major species dominating spontaneous MLF in wine (Berbegal et al., 2016). More importantly, *L. plantarum* can preferably adapt to the harsh wine environment, give less accumulation of biogenic amines, and retain more aromas (Wang et al., 2020). In addition, *L. plantarum* can produce antimicrobial compounds, which can inhibit the growth of other putrefaction microorganisms and reduce the use of SO_2_ (Lee et al., 2020). Moreover, *L. plantarum* has more enzyme genes that can produce wine aromas, or act on the aroma precursors in wine to release more rich and complex aroma compounds (Bokulich et al., 2015; Berbegal et al., 2016; Wang et al., 2020). Therefore, *L. plantarum* has attracted much more attention from researchers, and some *L. plantarum* strains have been commercially utilized as MLF starters in wine-making. Organic acid metabolism by lactic acid bacteria plays a significant role in cell growth, metabolism, and wine quality improvement. The co-metabolism of citric acid with glucose results in greater biomass and provides additional pathways for NADP^+^ regeneration and ATP production (Ramos and Santos, 1996). Citrate metabolism could improve wine aroma and increase aroma complexity, through the production of various volatile compounds, such as acetate, diacetyl, acetoin, and butanediol (Olguìn et al., 2009; Cappello et al., 2017). The 2-hydroxycarboxylate transporter family (2HCT) is a family of organic acid transporters found exclusively in the bacterial kingdom. They function in the metabolism of the di- and tricarboxylates (malate and citrate), mostly in fermentative pathways involving decarboxylation of malate or oxaloacetate. Detailed structural models of these transporters have been established in some species (Sobczak and Lolkema, 2005). The studies on the structure and function of 2HCT in LAB mainly focused on the citrate transporter (CitP) of *Leuconostoc mesenteroides* and the malate transporter (MleP) of *Lactococcus lactis* (Lolkema et al., 2005), but there are few reports on the 2HCT in the main strains of wine MLF. In the previous study, a strain of *L. plantarum* XJ25 with good MLF performance was isolated from wine, and its whole genome was sequenced and submitted to NCBI (accession number: NZ_CP068448). Through automated computational analysis using the gene prediction method of protein homology, it is concluded that the gene JKL54_04345 may encode citrate transporter (CitP).

Genetic manipulation is a key method for controlling the gene expression and studying the molecular mechanism of *L. plantarum.* In recent years, a number of expression vectors have been developed to express proteins in *Lactococcus lactis*, e.g., pMG36e. pMG36e has successfully expressed a number of proteins (Kim et al., 2006; Zhang et al., 2012; Liu et al., 2017). However, some reports have shown that the expression effect of some heterogenous proteins appeared unstable (Kim et al., 2009). The original pMG36e plasmid with erythromycin resistance gives some false-positive colonies when applied in the genetic manipulation of *Escherichia coli* (van de Guchte et al., 1989). Kanamycin, an antibiotic isolated from *Streptomyces kanamyceticus*, has been one of the most commonly used antibiotics because of its low cost and good therapeutic effect on the wide antibacterial spectrum (Liu et al., 2022). Huang et al. (2019) found the pHSP02 plasmid, in which sgRNA was driven by synthetic promoter P_11_, greatly outperformed the others in terms of editing efficiency (>89.4%) and mutant purity (100%).

Therefore, in this study, we first inserted the kanamycin resistance gene (*KanR*) from pLCNICK into pMG36e to obtain a double-resistant plasmid pMG36ek, and then replaced the P_32_ promoter with the synthetic promoter P_11_, in order to build a highly efficient expression vector pMG36ek11. Second, through gene knockout by pLCNICK based on CRISPR/Cas9 system and gene complement by the modified pMG36e, the function of the predicted citrate transporter gene JKL54_04345 (*citP*) was verified.

## Materials and Methods

### Bacterial Strains, Media, and Culture Conditions

The plasmid vectors used in this study were maintained in *Escherichia coli* strain DH5α (Tiangen, China) cultured in Luris-Bertani (LB) broth (Hopebio, China). *L. plantarum* XJ25 isolated from wine were propagated statically at 37°C in de Man, Rogosa, and Sharpe (MRS) medium (Hopebio, China). The fermentation experiments were performed in MRSg (a modified MRS medium containing yeast extract 4 g/L, K_2_HPO_4_ 2 g/L (NH_4_)_2_SO_4_ 2 g/L, NaCl 5 g/L, Tween-80 1 ml/L, MgSO_4_ **·**7H_2_O 0.2 g/L, MnSO_4_·4H_2_O 0.04 g/L, l-malic acid 1 g/L, citric acid 1 g/L, and pH 3.8 adjusted with HCl), MRSc (citric acid 2 g/L, l-malic acid 0 g/L, other components, and pH are the same as MRSg) and MRSm (l-malic acid 2 g/L, citric acid 0 g/L, other components and pH are the same as MRSg). The antibiotics were supplemented at the following concentrations when needed for plasmid maintenance: 50 μg/ml kanamycin for *E. coli,* and 50 μg/ml erythromycin for *L. plantarum*. All the strains used in this study were listed in Table 1.

**TABLE 1:** Bacterial strains and plasmid vectors used in this study.

Strain or plasmid	Genotype (description)	Source
Strains		
*E. Coli* DH5α	Commercial host for cloning	Laboratory
*L. plantarum* XJ25	Wild-type	This work
XJ25-Δ*citP*	*citP* knockout mutant	This work
XJ25-Δ*citP*-pMG36ek-*citP*	*citP* complement mutant with pMG36ek-*citP*	This work
XJ25-Δ*citP*-pMG36ek11-*citP*	*citP* complement mutant with pMG36ek11-*citP*	This work
Plasmids		
pLCNICK	Knockout vector	Song et al. (2017)
pLCNICK-Δ*citP*	*citP* knockout vector	This work
pMG36e	Shuttle vector with P_32_-promoter, *Emr*	van de Guchte et al. (1989)
pMG36ek	Shuttle vector with P_32_-promoter, *Emr*, and *KanR*	This work
pMG36ek11	Shuttle vector with P_11_-promoter, *Emr*, and *KanR*	This work
pMG36ek-*citP*	pMG36ek carrying *citP*	This work
pMG36ek11-*citP*	pMG36ek11 carrying *citP*	This work

### Construction of Plasmid Vectors and Mutants

Plasmid vectors used and constructed in this study are listed in Table 1. Plasmid vectors were constructed using standard molecular cloning, overlap extension PCR, and one-step cloning techniques. The primers used in PCR reactions are listed in Table 2. Restriction enzymes and DNA polymerases were purchased from Takara. PCR was performed with the C1000 Touch PCR System (Bio-Rad) using standard procedures. For genome editing of *L. plantarum* XJ25, gene JKL54_04345 as a citrate transport protein gene (*citP*) derived by automated computational analysis using gene prediction method of protein homology, was selected as a target.

**TABLE 2:** Primers used in this study.

Primer	Sequence (5′–3′)
citP-up-1	ctt​ttt​cta​aac​tag​ggc​ccA​TGT​GCA​GCA​CAC​ATT​TTT​GAT​G
citP-up-2	ATC​AGA​CTA​CAT​CTC​AAT​TCC​TCC​TCA​TAC​TTA​CTC
citP-down-1	ATT​GAG​ATG​TAG​TCT​GAT​TTT​AAG​CAT​AAA​AAC​AGG
citP-down-2	ccg​agt​cgg​tgc​ttt​ttt​tGC​ACA​TGA​TTA​TTA​CTT​ATC​C
sgRNA-1	aaa​aaa​agc​acc​gac​tcg​g
citP-sgRNA-2	gga​tga​tat​cac​ctc​tag​aCC​AGT​GTT​TCG​ATG​AAC​GCA​gtt​tta​gag​cta​gaa​ata​gc
citP-ha-1	TGA​TGA​GTA​AGT​ATG​AGG​AGG​AA
citP-ha-2	TGA​CCG​AAT​GGA​CAT​GCT​AT
citP-in-1	TGTTTGTGCGGCTGTTT
citP-in-2	GCATTGGGCGTATCTTTA
pLCNICK-test-1	aaa​agg​gat​agt​aat​tca​ttc​ctg​g
pLCNICK-test-2	tgcgagttgaccgtggg
citP-express-1	agg​taa​aaa​aat​att​cgg​agg​aat​ttt​gaa​ATG​ACA​CTA​AAT​AAG​GTC​AAG​TAT​CGT​GA
citP-express-2	aag​gtt​caa​aat​att​aaa​ttt​tac​cgg​tca​CTA​CCA​AAT​ACC​AAT​CAC​TTT​CAT​CCA​GA
pLCNICK-KanR-1	gct​cga​cat​act​gtt​ctt​cct​tag​aaa​aac​tca​tcg​agc​atc
pLCNICK-KanR-2	ttg​tga​atc​ggg​tcg​atc​ggg​gaa​agc​cac​gtt​gtg​tct​c
pMG36e-KanR-1	ccg​atc​gac​ccg​att​cac​aa
pMG36e-KanR-2	gga​aga​aca​gta​tgt​cga​gc
pMG36e-express-1	tga​ccg​gta​aaa​ttt​aat​att​ttg​aac​ctt
pMG36e-express-2	ttc​aaa​att​cct​ccg​aat​att​ttt​tta​cct
P11-1	tat​ggg​tcg​atc​gaa​ttc​AGC​GCT​ATA​GTT​GTT​GAC​AGA​ATG​GAC​ATA​CT
P11-2	ttc​aaa​att​cct​ccg​aat​AGC​AAC​ATT​ATA​TCA​TAG​TAT​GTC​CAT​TCT​GT
pMG36e-Promoter-1	attcggaggaattttgaa
pMG36e-Promoter-2	gaattcgatcgacccata
pMG36e-test-1	gca​cgg​tcg​atc​ttc​tat​at
pMG36e-test-2	tcg​caa​cag​aac​cgt​ttc​ta
16SrRNA-1	GCAACGAGCGCAACCC
16SrRNA-2	GACGGGCGGTGTGTAC
L-ldh-1	TGTGCCTCGTAAGCCTG
L-ldh-2	GCCCCCTTCTGACTAAT
citR-1	AGTAAGGCTTCGCTCTT
citR-2	TGA​CCG​AAT​GGA​CAT​GCT​AT

### Construction of *citP* Knockout Vector

The skeleton of pLCNICK was obtained by double digestion with *Xba*I and *Apa*I (Song et al., 2017). Two 1.0 kb fragments flanking *citP* (*citP*-up and *citP*-down) were amplified from *L. plantarum* XJ25 genomic DNA using the primers *citP*-up-1/*citP*-up-2 and *citP*-down-1/*citP*-down-2, respectively. A 122 bp sgRNA framework that targets *citP* (*citP*-sgRNA) was obtained by PCR using the primers sgRNA-1/*citP*-sgRNA-2 with pLCNICK as the template. This fragment was then assembled with *citP*-up and *citP*-down by overlap extension PCR, which yielded a new fragment, *citP*-uds. Subsequently, the backbone of pLCNICK and the fragment *citP*-uds were assembled to produce a new plasmid, pLCNICK-Δ*citP*, using the one-step cloning kit (Tiangen, China). Thereafter, positive clones were verified by PCR amplification with the primers pLCNICK-test-1 and pLCNICK-test-2.

### Construction of *citP* Overexpression Vectors From Original pMG36e

The original pMG36e vector was modified to add the *KanR* gene and improve the expression efficiency. pMG36e contains an erythromycin resistance gene *Emr*, a P_32_ promoter, a multiple cloning sites (MCS), the start of an open reading frame, and a *prtP* transcriptional terminator, and it is known to be a constitutive expression vector for the inserted gene in *Lactococcus lactis* (van de Guchte et al., 1989). We added a resistance gene to construct a new expression vector pMG36ek and then replaced a promoter to construct another new expression vector pMG36ek11. The vector was linearized by PCR amplification, and the multiclone site was completely replaced by the target gene *citP* to avoid the production of fusion protein affecting enzyme activity. Accordingly, two expression plasmids pMG36ek-*citP* and pMG36ek11-*citP* were constructed.

To generate plasmid pMG36ek, the backbone of pMG36e was obtained by PCR using primers pMG36e-KanR-1 and pMG36e-KanR-2, the *KanR* gene was amplified from pLCNICK using primers pLCNICK-KanR-1 and pLCNICK-KanR-2. Then, all the fragments were assembled to produce the plasmid pMG36ek.

Plasmid pMG36ek-*citP* was generated using two DNA fragments: the backbone of pMG36ek (amplified using primers pMG36e-express-1 and pMG36e-express-2), the *citP* fragment (obtained by PCR using primers citP-express-1 and citP-express-2 with *L. plantarum XJ25* genomic DNA as the template). The above two fragments are connected to form overexpression vector pMG36ek-*citP*.

In order to replace the P_32_ promoter with the P_11_ promoter (Rud et al., 2006), primers P11-1 and P11-2 were used to amplify the P_11_ fragment. Plasmids pMG36ek and pMG36ek-*citP* were linearized by PCR using primers pMG36e-promoter-1 and pMG36e-promoter-2 and then ligated with the P_11_ fragment, respectively, to construct overexpression vectors pMG36ek11 and pMG36ek11-*citP*.

Thereafter, positive clones were verified by PCR amplification with the primers pMG36e-test-1 and pMG36e-test-2.

### Transformation

Heat shock transformation was carried out according to the instructions of the competent cells of *E. coli* DH5α. After shaking at an appropriate temperature (knockout vector pLCNICK-Δ*citP* at 30°C, overexpression vectors pMG36ek-*citP*, and pMG36ek11-*citP* at 37°C) and 200 rpm for 1 h, the corresponding antibiotic plates were coated. A single colony was selected for colony PCR verification using primers pLCNICK-test-1 and pLCNICK-test-2, pMG36e-test-1, and pMG36e-test-2.

Then these vectors were delivered into the wild type XJ25 or the *citP* knockout mutant by electroporation. The preparation of electrocompetent cells and electrotransformation were performed as previously described (Yang et al., 2015; Huang et al., 2019). In brief, 1% (v/v) inoculum of the overnight culture was transferred into 80 ml fresh SGMRS medium (MRS with 0.75 m sorbitol and 1% glycine). The cells were collected by centrifugation at 4,000 rpm, 20 min for 4°C when OD_600_ reached 0.4–0.6. The cell pellets were washed twice with 1 mM MgCl_2_ and resuspended in 1 ml SM buffer (952 mM sucrose and 3.5 mM MgCl_2_). The competent cells were aliquoted and stored at -80°C. The electroporation was performed with Gene Pulser Xcell (Bio-Rad, United States) and 2 mm cuvette (BTX, United States) with the following parameters: 2 kV, 25 μF, 400 Ω. One milliliter of the recovery SMRS medium (MRS with 0.5 M sucrose, 0.1 M MgCl_2_) was added into a cuvette and the mixture was recovered within 2–3 h, and then plated on the MRS agar supplemented with erythromycin (Meng et al., 2021).

### Identification of Mutants

The screening of mutants was performed following the protocol as described previously with modifications (Wang et al., 2021). The transformant colonies of *L. plantarum* XJ25 were inoculated into the MRS agar plates with the addition of erythromycin. The plates were incubated anaerobically at 37°C until colonies were observed. Colony PCR (cPCR) was then performed to screen the putative mutants. Successful transformants were selected from the corresponding antibiotic plates. Positive clones were verified by PCR amplification with the primers *citP*-in-1 and *citP*-in-2 to obtain the mutants. The primers *citP*-ha-1 and *citP*-ha-2 were used to verify the genetic modification on the chromosome. The genome of *L. plantarum* XJ25 was used as the control. PCR products were sequenced to validate the knockout.

### Plasmid Curing

For continuous editing, the mutant was subcultured in the MRS medium for two generations, followed by streaking on the MRS agar plate. The single colonies were amplified with primers pLCNICK-test-1 and pLCNICK-test-2 to confirm the knockout plasmid curing.

### Quantitative Reverse Transcription-PCR

The wild type XJ25 and the mutants (XJ25-Δ*citP*, XJ25-Δ*citP*-pMG36ek-*citP*, and XJ25-Δ*citP*-pMG36ek11-*citP*) were incubated overnight in the MRS medium at 37°C. Then overnight cultures were inoculated into the fresh MRS medium with an inoculum volume of 5% (v/v). When the OD_600_ reached 1.0, the cells were harvested, and washed twice with the MRSg medium, then resuspended in the same volume of the MRSg medium. The resuspension was inoculated into the MRSg medium with an inoculum volume of 5% (v/v). After incubation at 37°C for 30 min, the cells were collected for RNA extraction.

RNA preparation and reverse transcription were performed using AG RNAex Pro Reagent and Evo M-MLV RT Kit with gDNA Clean for qPCR II (Agbio, China), respectively, according to the instructions described by the manufacturer. The concentration and quality of the RNA samples were determined using BioDrop μLite Spectrophotometer (BioDrop, England) before reverse transcription.

Quantitative PCR (qPCR) reactions were performed using SYBR Green Premix Pro Taq HS qPCR Kit (Agbio, China) by a CFX-96 system (Bio-Rad, United States). The primers of 16S rRNA, *citP*, *L-ldh*, and *citR* (Table 2) were used for the quantification of mRNA level of the reference gene, *citP* and adjacent up and downstream genes of *citP*. qPCR reactions were loaded in triplicate and assays were performed following the program proposed by the protocol of SYBR Green Premix Pro Taq HS qPCR Kit. The Ct (threshold value) calculated for *citP*, *L-ldh*, and *citR* were compared with values made from the calibration sample (16S rRNA). The values of these genes were normalized with that of 16S rRNA to estimate the relative copy numbers of the gene (Kiriya et al., 2017). The relative gene expression was calculated using the 2-^ΔΔCt^ method (Inwood et al., 2020).

### Evaluation of Organic Acid Metabolism by HPLC Analysis

The wild type (XJ25) and the mutants (XJ25-Δ*citP*, XJ25-Δ*citP*-pMG36ek-*citP*, and XJ25-Δ*citP*-pMG36ek11-*citP*) were incubated in the MRS medium until OD_600_ reached 1.0, then 5% (v/v) inoculum of this culture was transferred into the fresh MRSg medium, respectively. The cells were immediately recovered by centrifugation at 5000 × g for 5 min, then the fresh MRSg medium was added to the obtained bacteria, and after incubation at 37°C for 24 h, the supernatant solution was collected by centrifugation and filtering through a 0.45 µm filter membrane for further analysis. The samples were prepared in the MRSc and MRSm media using the same method.

Citric acid, l-malic acid, lactic acid, and acetic acid, in the samples, were analyzed by HPLC as previously described by Rossouw et al. (2012). The standards were purchased from Shanghai Aladdin Bio-Technology Co., Ltd (Aladdin, China). The organic acid contents of the samples s were detected with an HPLC system (1260 Infinity II, Agilent, United States) using a 300 × 7.8 mm i.d. Aminex HPX-87H Column (Bio-Rad, United States) with a column temperature of 60°C at a flow rate of 0.6 ml/min. The mobile phase was 5 mM H_2_SO_4_ prepared by diluting reagent-grade sulfuric acid with distilled water. Organic acids were detected at a wavelength of 210 nm. All analytical determinations were done in triplicate in each sample.

### Evaluation of Diacetyl and Acetoin Production by GC-MS Analysis

Diacetyl and acetoin in the samples were analyzed by GC-MS as previously described by Lin et al. (2019). The separation, detection, and quantification of volatile compounds were performed on a GC-MS system (5977B GC/MSD, Agilent, United States) coupled with PAL3 Series II autosampler systems (Agilent, United States) and equipped with an HP-INNOWAX capillary column (60 m × 0.25 mm inner diameter, 0.25 μm film thickness, Agilent, United States). The conditions of GC-MS in this study were applied as previously reported with some modifications (Zhao et al., 2021). Ultrapure helium was used as the carrier gas at 1.0 ml/min. The initial column temperature was set at 40°C for 3 min. Afterward, the temperature was raised to 160°C at a rate of 5°C/min, then 7°C/min to 230°C, and held at 230°C for 8 min. The mass detector conditions were as follows: The mass spectrum was acquired at 70 eV in electron ionization mode, scan range was 29–350 m/z, and scanning frequency in full scan mode was 4.4 times/s.

The volatiles were identified by matching the obtained mass spectra with the Wiley libraries and by comparing the retention indices (RI) to those of the compounds reported in the NIST 17 and the literature. According to the method proposed by Wu et al. (2016), the quantification procedure was carried out with the internal standard quantification method with light modification. 4-Methyl-2-pentanol was employed as the internal standard compound.

### Statistical Analysis

The data were reported as means ± standard deviation of three triplicates and were analyzed statistically by one-way ANOVA. Means were compared by Duncan’s multiple range tests. The differences with *p*-values < 0.05 were considered statistically significant. The statistical software utilized was SPSS 19.0 (SPSS, United States). The experimental results were analyzed using GraphPad Prism (GraphPad, United States).

## Results

### Construction of *citP* Knockout Mutant

**FIGURE 1:**
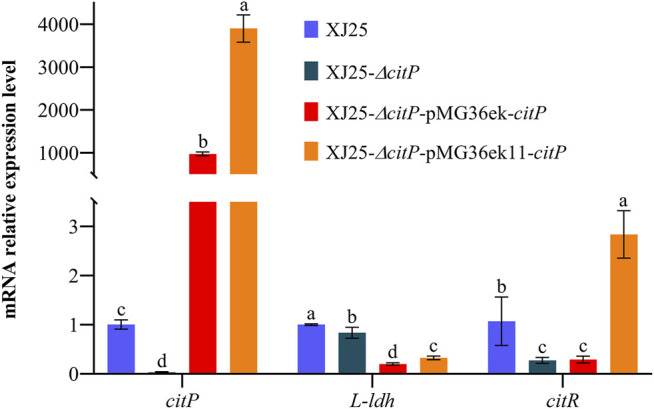
Determination of citP, L-ldh, and citR gene expression levels in the wild-type XJ25 and the mutants. Data are expressed as mean ± standard deviation (*n* = 3). a-d means with different lower-case letters in the same row indicate significant differences (*p* < 0.05).

The map of *citP* knockout vector used in this work is shown in Supplementary Figure S1A. The knockout vector pLCNICK-Δ*citP* was verified using PCR and sequencing. The sequencing of the PCR amplicon confirmed the precise knockout of *citP* as expected (Supplementary Figure S1B). The length of the *citP* knockout vector corresponded to the expected size of 14365 bp. The verified knockout vector was transformed into XJ25 to construct the *citP* deletion strains. All of the strains showed the expected electrophoretic bands (418 bp for *citP*). One *citP* knockout mutant (land 20), XJ25-Δ*citP*, was obtained, which was obviously distinguishable from the wild types (land 01) (Supplementary Figure S2A). These results suggested that *citP* knockout mutant was constructed successfully. To further validate the knockout and analysis the effect of *citP* knockout on other related genes, together with *citP*, *L-ldh*, and *citR* genes were chosen and analyzed by RT-qPCR in the MRSg medium (Figure 1). Compared with the relative expression levels of *citP* in the wild type XJ25, that in XJ25-Δ*citP* was reduced to 0.03-fold, demonstrating that the *citP* gene was removed in XJ25-Δ*citP*. The data also show that the expression levels of the *L-ldh* and *citR* genes in XJ25-Δ*citP* were lower than those in the wild-type XJ25. Compared with the wild-type XJ25, 0.83-fold and 0.27-fold decreases were found in XJ25-Δ*citP* for the expression levels of *L-ldh* and *citR*, respectively. These may suggest that *citP* positively influences the transcription of the citric acid metabolism-related genes.

### Construction of *citP* Complement Mutants

The construction process of *citP* overexpression vectors in this work is shown in Supplementary Figure S3. Summarily, in this study, two modified plasmid vectors, pMG36ek and pMG36ek11, were used to construct *citP* complement mutants derived from the *citP* knockout mutant XJ25-Δ*citP*. A kanamycin resistance gene (*KanR*) from pLCNICK was inserted into the original vector pMG36e to form pMG36ek, and then the P_32_ promoter of pMG36ek was replaced with the synthetic promoter P_11_ to form pMG36ek11. The length of these *citP* overexpression vectors corresponded to the expected size of 5717 bp or 5612 bp. The verified *citP* overexpression vectors were transformed into XJ25-Δ*citP* to construct the complement mutants. Through PCR verification described in Supplementary Figure S2B, all of the strains showed the expected electrophoretic bands, which were obviously distinguishable from XJ25-Δ*citP*. Compared to land 05 in XJ25-Δ*citP,* land 02, 08, and land 11 had the bright strip. These results indicate that *citP* complement mutants were successfully constructed using the pMG36ek and pMG36ek11 vectors. *citP* could be expressed successfully in XJ25-Δ*citP*. As shown in Figure 1, the relative expression levels of *citP* in the *citP* overexpression mutants XJ25-Δ*citP*-pMG36ek-*citP* and XJ25-Δ*citP*-pMG36ek11-*citP* mutants were much higher than that in the wild type XJ25 in the MRSg medium. Compared with XJ25*,* 973.77-fold and 3900.47-fold increase was found in XJ25-Δ*citP*-pMG36ek-*citP* and XJ25-Δ*citP*-pMG36ek11-*citP*, respectively, for the expression level of *citP*. For the expression level of *L-ldh*, a 0.20-fold decrease was found in XJ25-Δ*citP*-pMG36ek-*citP* and a 0.32-fold decrease was found in XJ25-Δ*citP*-pMG36ek11-*citP*, compared with the wild-type XJ25. For the expression level of *citR,* it showed a 0.29-fold decrease in XJ25-Δ*citP*-pMG36ek-*citP* and a 2.84-fold increase in XJ25-Δ*citP*-pMG36ek11-*citP*, compared with the wild-type XJ25. Moreover, the relative expression levels of gene *citP, L-ldh*, and *citR* genes in XJ25-Δ*citP*-pMG36ek11-*citP* were 4.01, 1.62, 9.80-fold higher than that in XJ25-Δ*citP*-pMG36ek-*citP* (*p* < 0.05), respectively, indicating a significant difference in expression efficiency between the two vectors.

### Effect of *citP* Knockout and Complement on Organic Acid Metabolism

**FIGURE 2:**
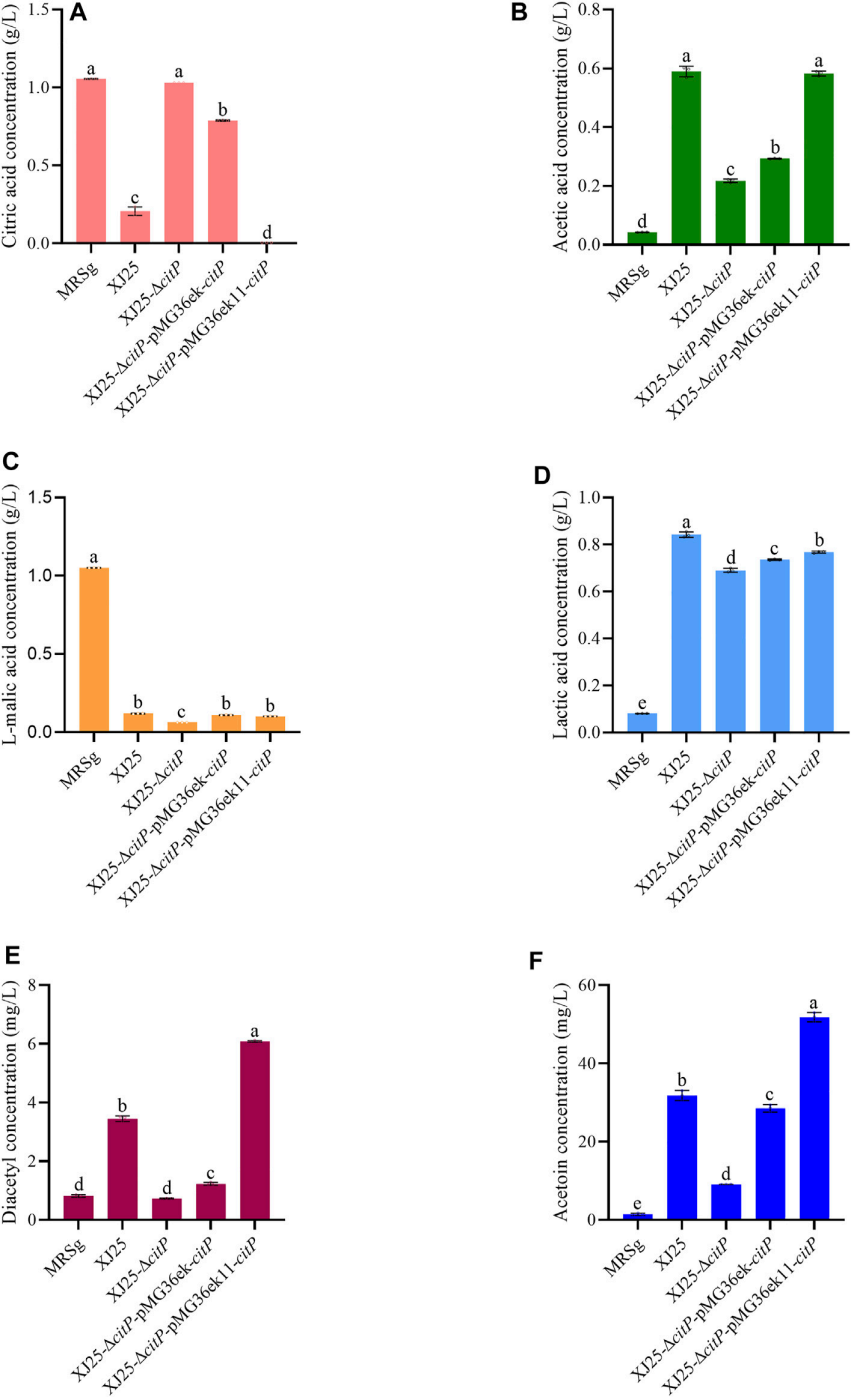
Changes in metabolite concentration in the MRSg medium after 24 h culture with the wild-type and the mutants **(A)** citric acid, **(B)** acetic acid, **(C)** L-malic acid, **(D)** lactic acid, **(E)** diacetyl and **(F)** acetoin. Data are expressed as mean ± standard deviation and (*n* = 3). a-e means with different lower-case letters in the same row indicate significant differences (*p* < 0.05).

The changes in organic acid concentration in MRSg medium after 24 h culture with the wild-type and the mutants are shown in Figure 2. Most of the citric acid in the culture medium (0.21 g/L residual citric acid) was used up by the wild-type XJ25 after 24 h culture (Figure 2A). However, the citric acid concentration (1.03 g/L) in the culture medium after 24 h culture with *citP* knockout mutant XJ25-Δ*citP* showed no significant differences from that in the original MRSg medium (1.06 g/L), suggesting XJ25-Δ*citP* strain could not utilize citric acid. The citric acid concentration in the culture medium for two *citP* complement mutants, XJ25 (0.21 g/L), XJ25-Δ*citP*-pMG36ek-*citP* (0.79 g/L), and XJ25-Δ*citP*-pMG36ek11-*citP* (0.00 g/L), were much lower than that for XJ25-Δ*citP,* suggesting that XJ25-Δ*citP*-pMG36ek-*citP* could not make full use of citric acid (25.47% utilization rate), and XJ25-Δ*citP*-pMG36ek11-*citP* could make full use of citric acid (100.00% utilization rate). The utilization rate of citric acid for XJ25-Δ*citP*-pMG36ek11-*citP* was 3.95 times higher than that for XJ25-Δ*citP*-pMG36ek-*citP* (*p* < 0.05).

For acetic acid production (Figure 2B), there was no significant differences between wild-type XJ25 and complement mutant XJ25-Δ*citP*-pMG36ek11-*citP*. The acetic acid concentration for XJ25-Δ*citP*-pMG36ek11-*citP* (0.58 g/L) was higher than that for XJ25-Δ*citP*-pMG36ek-*citP* (0.29 g/L) (*p* < 0.05). In addition, the acetic acid concentration for XJ25 (0.59 g/L) were much higher than that for XJ25-Δ*citP* (0.22 g/L). The acetic acid concentration for XJ25-Δ*citP* was 0.37-fold lower than that for the wild type XJ25. Additionally, a 1.99-fold increase in the acetic acid concentration was found in XJ25-Δ*citP*-pMG36ek11-*citP*, compared with XJ25-Δ*citP*-pMG36ek-*citP*.

The changes in l-malic acid concentration are shown in Figure 2C. No obvious differences in l-malic acid content were observed in the medium cultured with XJ25 (0.12 g/L), XJ25-Δ*citP*-pMG36ek-*citP* (0.11 g/L), and XJ25-Δ*citP*-pMG36ek11-*citP* (0.10 g/L). The lowest concentration of l-malic acid was observed in the medium cultured with XJ25-Δ*citP* (0.06 g/L)*,* while the highest one was observed in the original MRSg medium (1.05 g/L). The l-malic acid concentration for XJ25-Δ*citP* was 0.53-fold lower than that for XJ25.

As shown in Figure 2D, the production of lactic acid was significantly different between XJ25 and XJ25-Δ*citP* (*p* < 0.05). XJ25-Δ*citP* (0.69 g/L) displayed a lower yield of lactic acid than XJ25 (0.84 g/L), XJ25-Δ*citP*-pMG36ek-*citP* (0.74 g/L), and XJ25-Δ*citP*-pMG36ek11-*citP* (0.77 g/L)*.* The lactate production of the complement mutants was lower than that of the wild-type, which was consistent with the results of *L-ldh* expression (Figure 1).

**FIGURE 3:**
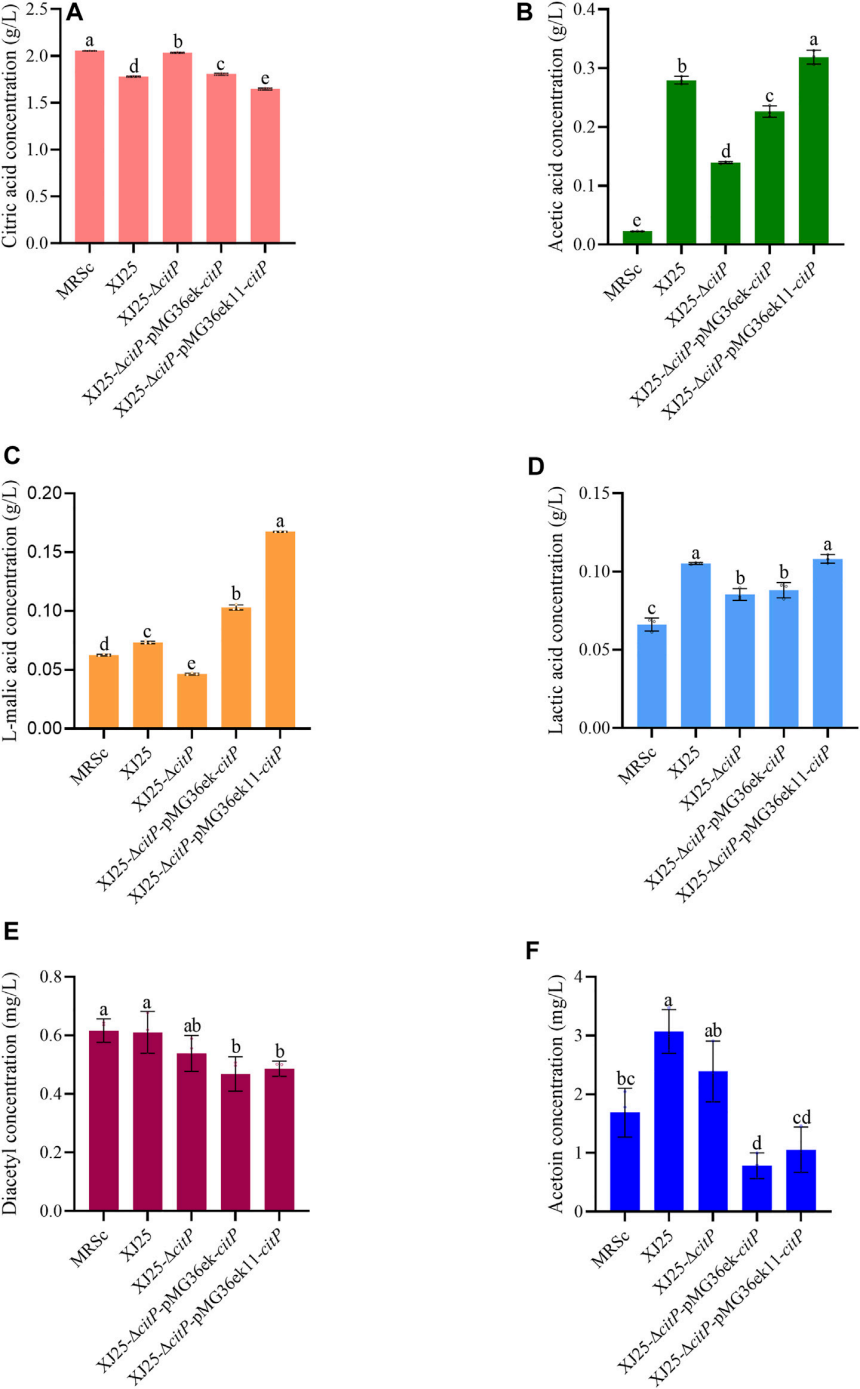
Changes in metabolite concentration in the MRSc medium after 24 h culture with the wild-type and the mutants **(A)** citric acid, **(B)** acetic acid, **(C)** L-malic acid, **(D)** lactic acid, **(E)** diacetyl and **(F)** acetoin. Data are expressed as mean ± standard deviation (*n* = 3). a-e means with different lower-case letters in the same row indicate significant differences (*p* < 0.05).

In the MRSc medium only containing 2.06 g/L citric acid, XJ25-Δ*citP* still could barely utilize citric acid (2.03 g/L) (Figure 3A). The wild-type XJ25 (1.78 g/L), XJ25-Δ*citP*-pMG36ek-*citP* (1.81 g/L), and XJ25-Δ*citP*-pMG36ek11-*citP* (1.65 g/L) could utilize citric acid; however, the utilization rate of citric acid decreased significantly compared with that in the MRSg medium, and the highest utilization rate of citric acid found in XJ25-Δ*citP*-pMG36ek11-*citP* was only 19.73% after 24 h incubation. Compared with the l-malic acid concentration in the original MRSc medium (0.06 g/L), a slight decrease of l-malic acid concentration was found in the medium cultured with XJ25-Δ*citP* (0.05 g/L); however, the l-malic acid concentration in the medium cultured with XJ25, XJ25-Δ*citP*-pMG36ek-*citP*, and XJ25-Δ*citP*-pMG36ek11-*citP* increased to 0.07 g/L, 0.10 g/L and 0.17 g/L, respectively (Figure 3C). The production tendency of lactic acid and acetic acid of these strains in the MRSc medium was almost consistent with that in the MRSg medium (Figures 3B,D). The productions of lactic acid and acetic acid by XJ25-Δ*citP* were obviously lower than that by the wild-type XJ25 and the two complement mutants.

**FIGURE 4:**
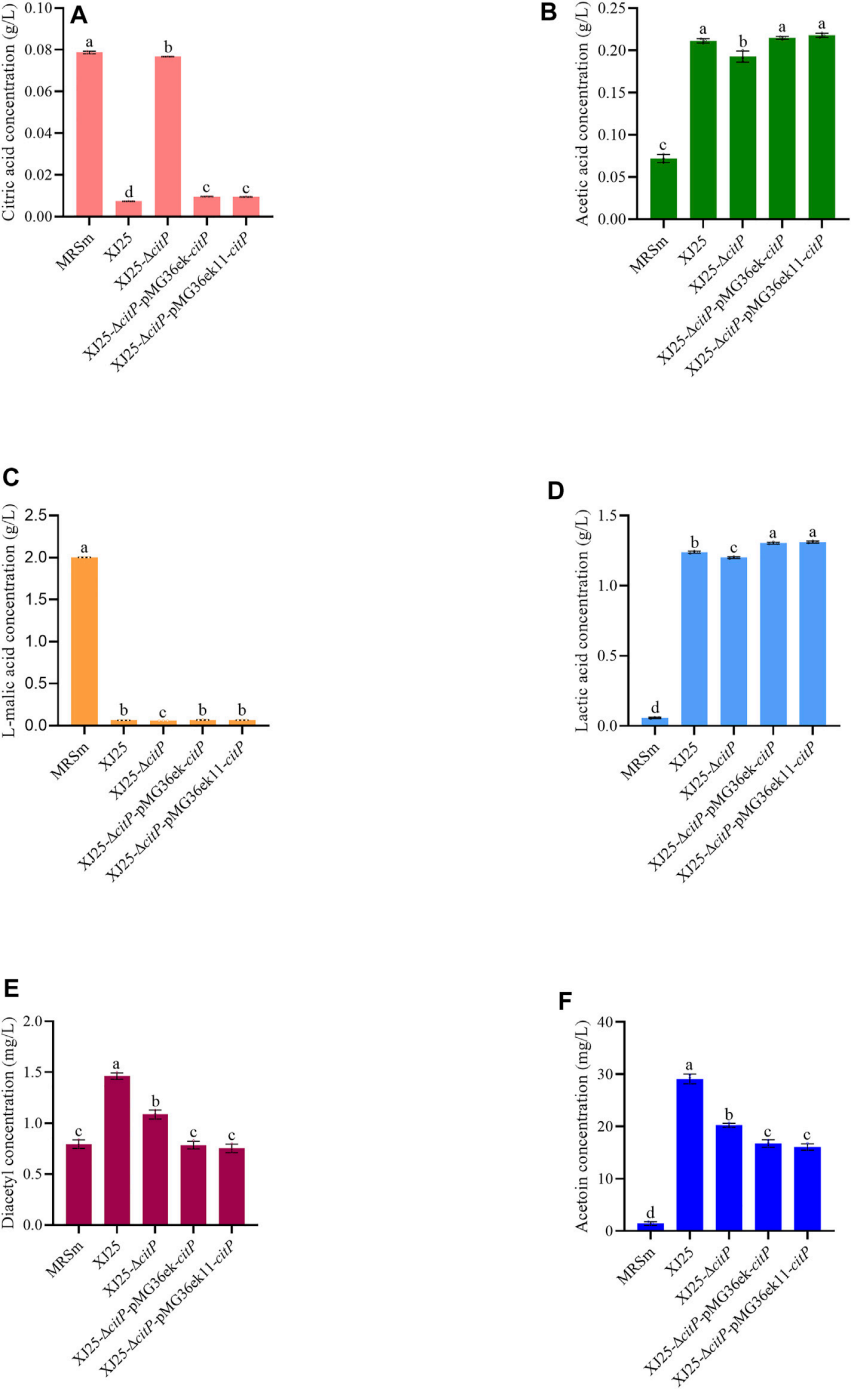
Changes in metabolite concentration in the MRSm medium after 24 h culture with the wild-type and the mutants **(A)** citric acid, **(B)** acetic acid, **(C)** L-malic acid, **(D)** lactic acid, **(E)** diacetyl and **(F)** acetoin. Data are expressed as mean ± standard deviation (*n* = 3). a-e means with different lower-case letters in the same row indicate significant differences (*p* < 0.05).

In the MRSm medium only containing 2.00 g/L l-malic acid, all the strains could fully utilize l-malic acid (Figure 4C). Though the very low citric acid concentration in the MRSm medium (0.08 g/L), the wild type XJ25 (0.01 g/L), XJ25-Δ*citP*-pMG36ek-*citP* (0.01 g/L), and XJ25-Δ*citP*-pMG36ek11-*citP* (0.01 g/L) could almost use up the citric acid, and XJ25-Δ*citP* (0.08 g/L) almost could not utilize the citric acid (Figure 4A). Similar to the situation in the MRSg and MRSc media, XJ25-Δ*citP* gave the lowest concentration of lactic acid and acetic acid in the MRSm medium (Figures 4B,D).

### Effect of *citP* Knockout and Complement on Diacetyl and Acetoin Production

The changes in diacetyl and acetoin concentration in the MRSg medium after 24 h culture with the wild-type and the mutants are listed in Figure 2. XJ25-Δ*citP* did not produce diacetyl after 24 h culture (Figure 2E). The diacetyl production of XJ25 (2.63 mg/L) after a 24 h culture was obviously higher than that of XJ25-Δ*citP*-pMG36ek-*citP* (0.42 mg/L) but obviously lower than that of XJ25-Δ*citP*-pMG36ek11-*citP* (5.27 mg/L). The production of diacetyl for XJ25-Δ*citP*-pMG36ek11-*citP* was 12.64-fold higher than that for XJ25-Δ*citP*-pMG36ek-*citP*.

In the case of acetoin, the production in the MRSg medium (Figure 2F), the trend was basically similar to diacetyl. The acetoin production of XJ25 (30.36 mg/L) after a 24 h culture was slightly higher than that of XJ25-Δ*citP*-pMG36ek-*citP* (27.07 mg/L) but obviously lower than that of XJ25-Δ*citP*-pMG36ek11-*citP* (50.41 mg/L). The production of acetoin for XJ25-Δ*citP*-pMG36ek11-*citP* was 1.86-fold higher than that for XJ25-Δ*citP*-pMG36ek-*citP*. The only difference was that XJ25-Δ*citP* also yield 7.64 mg/L acetoin.

In the MRSc media, the diacetyl and acetoin production of XJ25-Δ*citP* showed no significant difference with that of XJ25; however, the diacetyl and acetoin production of the two complement mutants were obviously lower than that of XJ25 (Figures 3E,F). In the MRSm medium, the highest production of diacetyl and acetoin was found in XJ25, followed by XJ25-Δ*citP* and the two complement mutants (Figures 4E,F).

## Discussion

In our study, a predicted citrate transporter gene JKL54_04345 (*citP*) was knocked out successfully and efficiently in *L. plantarum* XJ25 using the CRISPR editing plasmid pLCNICK. However, the previous studies have reported that pLCNICK can effectively edit the genome of *Lactobacillus casei* LC2W but cannot edit the genome of *L. plantarum* WCFS1 (Song et al., 2017; Huang et al., 2019). This may be due to the differences in Cas9-mediated genome editing methods or the selected strains (Leenay et al., 2019). The homologous transporters CitP have been reported with the functions in citrate utilization and by LAB such as *Leuconostoc mesenteroides* and *Lactococcus lactis* (MartyTeysset et al., 1996; Pudlik and Lolkema, 2011). CitP also catalyzes uptake of citrate in exchange with lactate during citrate-glucose co-metabolism (Pudlik and Lolkema, 2012). They have been verified as precursor/product exchangers in the 2HCT family that can couple the uptake of the substrate (the precursor, citrate) to the excretion of the end product (such as lactate). After multiple sequence alignment, it was found that the sequence similarity between CitP in *L. plantarum* and the homologs of 2HCT in other LAB reached 34.1–36.5%. (Supplementary Figure S4). Furthermore, most of the 2HCT genes in these LABs are located on the plasmid (David et al., 1990; Bandell et al., 1997; Sobczak and Lolkema, 2005), while *citP* of *L. plantarum* is located on the genome (NCBI accession number: NZ_CP068448). To verify the function of the predicted citrate transporter gene JKL54_04345 (*citP*) in *L. plantarum,* we constructed the *citP* knockout mutant (XJ25-Δ*citP*) using pLCNICK vector and the *citP* supplement mutants (XJ25-Δ*citP-*pMG36ek-*citP* and XJ25-Δ*citP-*pMG36ek11-*citP*) using two modified pMG36e vector.

Our results show that *citP* knockout in the wild-type *L. plantarum* XJ25 resulted in a loss of the ability of citric acid utilization; however, *citP* complement using pMG36ek11 vector in XJ25-Δ*citP* mutant recovered its ability to utilize citric acid (Figures 2A, 3A, 4A). Meanwhile, *citP* knockout and complement barely affected the utilization of l-malic acid by the mutants (Figures 2C, 3C, 4C). These indicated that this gene functioned as a citrate transporter and was the only gene responsible for the citrate transporter.

The metabolism of organic acid by LAB, especially for citric acid and malic acid, plays a significant role in wine-making, due to its contribution to organoleptic properties and quality of wine (Ali et al., 2010; Silva et al., 2015; Robles et al., 2019). After uptake by *CitP* from the medium, citrate can be converted to various metabolic end products, such as acetate, lactate, diacetyl, acetoin, and butanediol, which are very important for the aroma and complexity of wine (Olguin et al., 2009). As is expected, *citP* knockout significantly led to the reduction of acetate, lactate, diacetyl and acetoin production, and then *citP* complement significantly recovered the production of these compounds (Figures 2–4). Moreover, the production of the end products by these strains, including acetic acid, diacetyl and acetoin, was significantly positively associated with the utilization of citric acid, also indicating the important function of *citP* as citrate transporter. The only exception is lactic acid, the production of which showed slight differences among these strains.

Most of LAB utilize malic acid through the malolactic enzyme, to directly generate lactic acid and CO_2_, without any free intermediates (Mendes Ferreira and Mendes-Faia, 2020). l-malic acid can provide a strong and sharp taste sensation, but high level can result in a pungent taste (Robles et al., 2019). In our study, l-malic acid was almost used up by all strains, and the l-malic acid utilization in XJ25-Δ*citP* was slightly more than that in other strains (Figures 2C, 3C, 4C), illustrating that in addition to *citP*, *L. plantarum* XJ25 have other types of 2HCT for malate transport. Furthermore, the increased range of lactic acid was closely consistent with the decrease of l-malic acid, in agreement with the results of Li R.-Y. et al. (2018). Compared with XJ25, the more consumption of l-malic acid in XJ25-Δ*citP* but the lower production of lactic acid also verified the generated lactic acid was partially from citrate metabolism. In addition, in the MRSc medium, no consumption of malic acid in XJ25 and the two complement mutants but the increased production of lactic acid could also be an evidence for this speculation. These could explain for the exception in the previous paragraph.

A very interesting phenomenon was that in presence of malic acid, the utilization rate of citric acid by XJ25 was very high (80.50%) (Figure 2A); however, in the absence of malic acid, the utilization rate of citric acid by XJ25 sharply decreased (only 13.34%) (Figure 3A). It is previously reported that in *Lactococcus* species, the citrate fermentation is dependent on glycolysis, because glycolysis could provide the end product, lactate, in exchange for citrate from the medium by CitP (Sobczak and Lolkema, 2005). This process is called citrate-glucose co-metabolism, which is a secondary metabolic energy-generating route that produces proton motive force. Our results showed that in the presence of both malate and citrate, there existed citrate-malate co-metabolism in *L. plantarum*, during which the extracellular citrate also could be rapidly imported in exchange with the intracellular lactate formed from malate metabolism. This may also explain the phenomenon that during MLF in wine, the metabolism of citric acid is usually sequential to malic acid (Bartowsky and Henschke, 2004).

As a volatile acid, acetic acid is an unfavorable compound in wine, and its content needs to be strictly controlled during wine-making. *citP* knockout gave an almost 63.09% decrease in acetic acid production (Figure 2B), which indicates that the acetic acid is mainly from citrate metabolism and provides a possible strategy to decrease volatile acid content during MLF by reducing citric acid utilization.

Diacetyl, as an important flavor compound with a very low sensory threshold concentration (0.1 mg/L), is also produced by some other LAB genera, including *Leuconostoc, Lactobacillus*, and *Oenococcus* (Bartowsky and Henschke, 2004). Diacetyl donates a desirable buttery, nutty, or toasty aroma in wine at low concentrations (<5 mg/L); however, overmuch diacetyl in wine brings a caramellike off-odor. Acetoin is also an important flavor compound with a yogurt odor and a fatty creamy butter aroma (Dai et al., 2015). However, the sensory threshold of acetoin (15 mg/L) is significantly higher than that of diacetyl, and, during wine-making, it is usually difficult to reach the sensory threshold for acetoin. The formation of diacetyl and acetoin is closely linked to the citrate metabolism of LAB (Sternes et al., 2017; Li P. et al., 2018). Citrate metabolism gives pyruvate, and pyruvate gives α-acetolactate by α-acetolactate synthase. Then the nonoxidative decarboxylation and the nonenzymatic oxidative decarboxylation of α-acetolactate give acetoin and diacetyl, respectively. The reduction of diacetyl also gives acetoin (Mendes Ferreira and Mendes-Faia, 2020). Therefore, *citP* knockout turned off the uptake of citrate, which sharply decreased the production of α-acetolactate and consequently decreased the production of diacetyl and acetoin (Figures 2E,F). However, XJ25-Δ*citP* did not produce diacetyl but produced a certain amount of acetoin. The reason for this speculation is that the metabolism of pyruvate from other sources, such as malate metabolism, also gave acetoin, and the anaerobic environment in medium limited the formation of diacetyl from α-acetolactate and promoted the reduction of diacetyl to acetoin. The high yield of acetoin (18.78 mg/L) but almost no consumption of citric acid by XJ25-Δ*citP* in the MRSm medium could also support this speculation, in which a small part of malate was metabolized into pyruvate by malate dehydrogenase (EC 1.1.1.38) or malic enzyme (EC 1.1.1.39), and the subsequent pyruvate metabolism gave acetoin.

*L-ldh* and *citR* are two genes structurally adjacent to *citP* in the same cluster and annotated with the functions closely related to citrate metabolism. L-LDH (l-lactate dehydrogenase) plays a key role in the conversion of pyruvate to lactate in *Enterococcus faecalis* (Jonsson et al., 2009). *citR* is involved in the citrate metabolism as a transcriptional regulator (Sobczak and Lolkema, 2005). It is worth mentioning that the relative expression levels of *L-ldh* and *citR* genes in XJ25-Δ*citP* were much lower than in XJ25 (Figure 1). One reason we speculated for this phenomenon is that the loss of citric acid uptake in XJ25-Δ*citP* indirectly influenced the expression of the subsequent citrate-metabolism related genes, another reason we speculated is that *citP* knockout could structurally impact the expression of its nearby genes to some extent. This speculation could be verified by the fact that the complement mutant XJ25-Δ*citP*-pMG36ek-*citP* could not make full use of citric acid in the MRSg medium, even though its *citP* expression level was much higher than XJ25 (Figures 1, 2A).

As described above, two modified vectors (pMG36ek and pMG36ek11) from the original pMG36e vector as abovementioned were used in our study to complement *citP* in the *citP* knockout mutant. The only difference between pMG36ek and pMG36e was that pMG36ek had an additional kanamycin resistance gene, with the purpose to reduce the false-positive colonies screened by erythromycin during genetic manipulation. pMG36ek11 with the replaced synthetic promoter P_11_ show an extremely significant increase in expression efficiency of the targeted gene (Figure 1). Meanwhile, the complement mutant with pMG36ek11 gave significantly increased utilization of citric acid and generation of the metabolic end products (acetic acid, diacetyl, and acetoin) (Figure 2). These indicate that the modified pMG36ek11 can be used as a high-level expression vector applied in *L. plantarum*.

## Conclusion

This study verified the function of a predicted citrate transporter gene JKL54_04345 (*citP*) in *L. plantarum* for the first time. *citP* knockout mutant could not utilize citric acid at all; however, *citP* complement mutant recovered the ability of citric acid utilization. Meanwhile, the knockout and complement of *citP* barely affected the utilization of L-malic but remarkably affected the production of metabolic end products of citrate utilization. Therefore, it was verified that *citP* functioned as a citrate transporter and was the only gene responsible for citrate transporter in *L. plantarum*. In addition, the modified vector pMG36ek11 possesses a high-level expression efficiency in *L. plantarum* and shows a great application potential in LAB expression systems.

## Data Availability

The original contributions presented in the study are included in the article/Supplementary Material, further inquiries can be directed to the corresponding authors.
