# Supplementary figures and images for: Functional Verification of the Citrate Transporter Gene in a Wine Lactic Acid Bacterium, Lactiplantibacillus plantarum
Source: Front Bioeng Biotechnol. 2022 May 9;10:894870. doi: 10.3389/fbioe.2022.894870 (PMC9124760; doi:10.3389/fbioe.2022.894870)

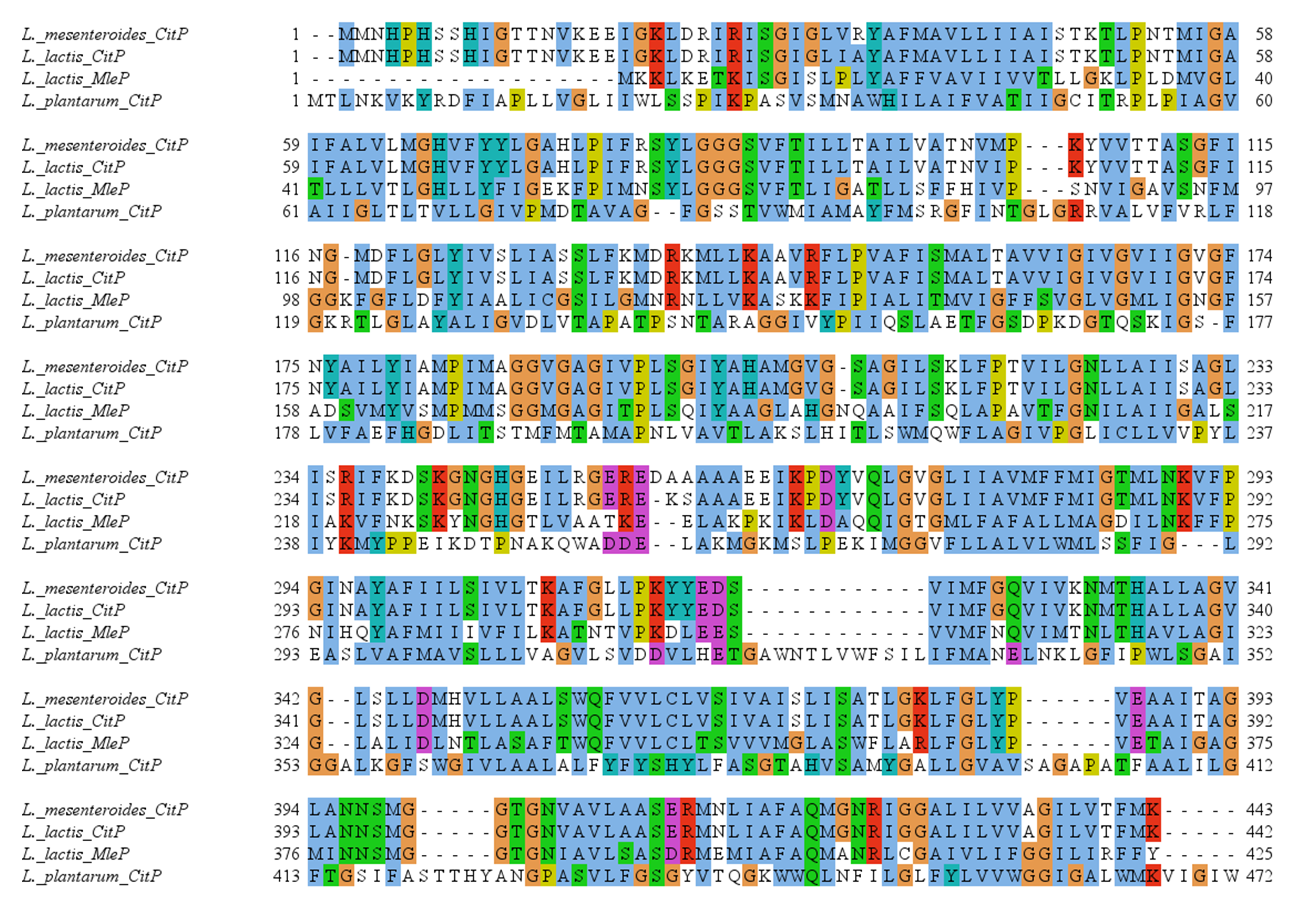

Supplement: Supplementary file 2 [file Image4.TIF]

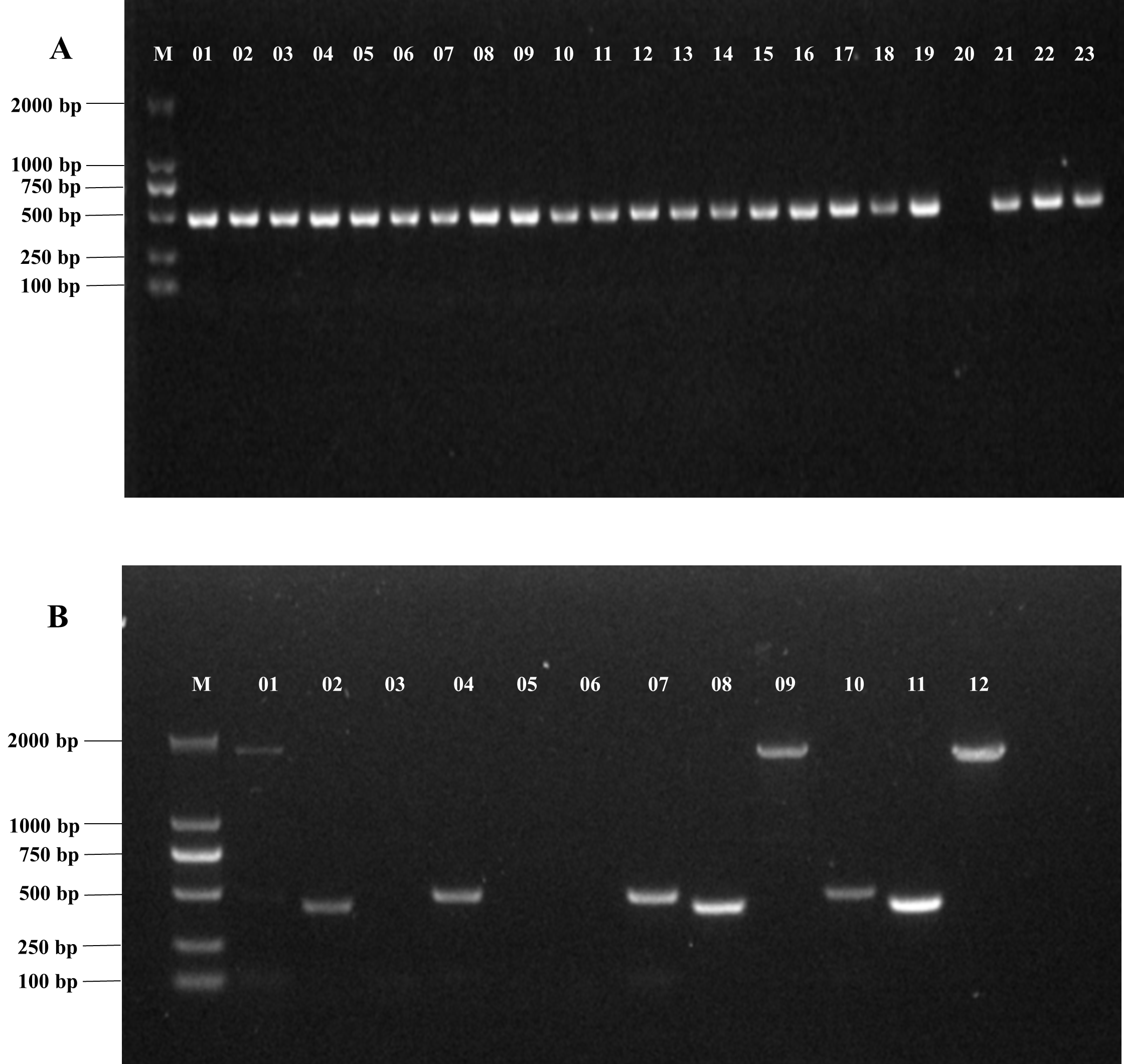

Supplement: Supplementary file 3 [file Image2.TIF]

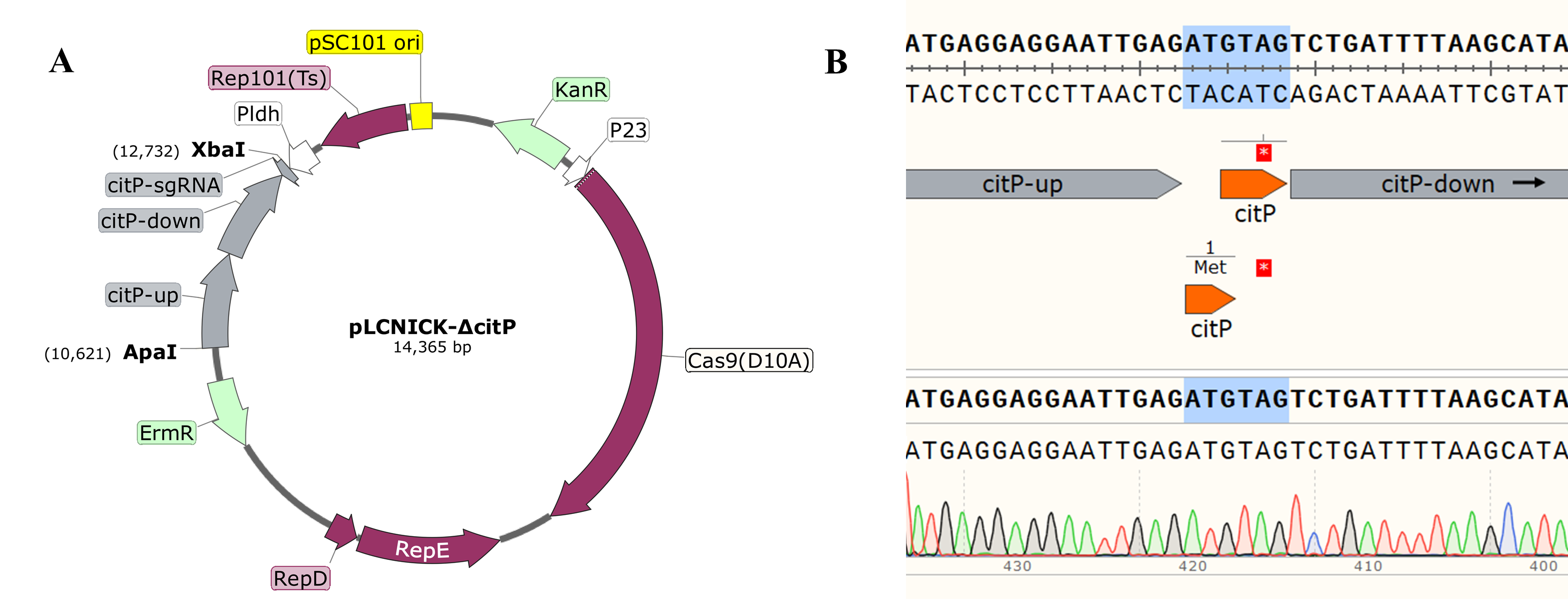

Supplement: Supplementary file 4 [file Image1.TIF]
